# Supplementary material for: Testing the effectiveness of different wash protocols to remove body surface contaminants in invertebrate food web studies
Source: PeerJ. 2023 Nov 21;11:e16018. doi: 10.7717/peerj.16018 (PMC10668814; doi:10.7717/peerj.16018)
Supplement: Supplemental Information 1 [file peerj-11-16018-s001.docx]

# Supplementary material:

**Suppl. Table 1:** List of used reagents with their molecular formula and manufacturer information

| Reagent | Molecular formula | Manufacturer |
| --- | --- | --- |
| Acetone | C_3_H_6_O | VWR, Darmstadt, Germany |
| Bleach 10% | NaClO | ITW Reagents, AppliChem, Darmstadt, Germany |
| Formaldehyde 37 % | CH_2_O | Merck, Darmstadt, Germany |
| Hydrogen peroxide 30 % | H_2_O_2_ | Carl Roth, Karlsruhe, Germany |
| Peracetic acid 2 % | C_2_H_4_O_3_ | Carl Roth, Karlsruhe, Germany |
| Sodium dodecyl sulfate, SDS 0.1 % | NaC_12_H_25_SO_4_ | Merck, Darmstadt, Germany |
| Sterillium® |  | Bode Chemie, Hamburg, Germany |
